# Use of polyvinylpyrrolidone-iodine solution for sterilisation and preservation improves mechanical properties and osteogenesis of allografts

**DOI:** 10.1038/srep38669

**Published:** 2016-12-09

**Authors:** Yantao Zhao, Xiantong Hu, Zhonghai Li, Fuli Wang, Yang Xia, Shuxun Hou, Hongbin Zhong, Feimin Zhang, Ning Gu

**Affiliations:** 1 Beijing Engineering Research Center of Orthopaedic Implants, First Affiliated Hospital of CPLA General Hospital, Beijing 100048, P. R. China; 2 Jiangsu Key Laboratory of Oral Diseases, Nanjing Medical University, Nanjing 210029, P. R. China; 3 State Key Laboratory of Bioelectronics, Jiangsu Key Laboratory of Biomaterials and Devices, School of Biological Science and Medical Engineering, Southeast University, Nanjing 210096, P. R. China; 4 Suzhou Institute & Collaborative Innovation Center of Suzhou Nano Science and Technology, Southeast University, Suzhou 215000, P. R. China

## Abstract

Allografts eliminate the disadvantages associated with autografts and synthetic scaffolds but are associated with a disease-transmission risk. Therefore, allograft sterilisation is crucial. We aimed to determine whether polyvinylpyrrolidone-iodine (PVP-I) can be used for sterilisation and as a new wet-preservation method. PVP-I–sterilised and preserved allografts demonstrated improved mechanical property, osteogenesis, and excellent microbial inhibition. A thigh muscle pouch model of nude mice showed that PVP-I–preserved allografts demonstrated better ectopic formation than Co_60_-sterilised allografts (control) *in vivo* (P < 0.05). Furthermore, the PVP-I–preserved group showed no difference between 24 h and 12 weeks of allograft preservation (P > 0.05). PVP-I–preserved allografts showed more hydrophilic surfaces and PVP-I–sterilised tendons showed higher mechanical strength than Co_60_-sterilised tendons (P < 0.05). The level of residual PVP-I was higher without washing and with prolonged preservation (P < 0.05). *In vitro* cellular tests showed that appropriate PVP-I concentration was nontoxic to preosteoblast cells, and cellular differentiation measured by alkaline phosphatase activity and osteogenic gene markers was enhanced (P < 0.05). Therefore, the improved biological performance of implanted allografts may be attributable to better surface properties and residual PVP-I, and PVP-I immersion can be a simple, easy method for allograft sterilisation and preservation.

In cases of vital damages, allografting of tissues such as bone, tendon, and meniscus is currently an alternative to autografting, as the former has several advantages including lower surgical morbidity, less surgical time, and better cosmetic appearance^1^. Its utilisation for orthopaedic procedures has increased steadily over the past decade, with approximately one million allografts transplanted annually^2^. This number is estimated to increase more dramatically in in the near future^3^. Allografts eliminate the disadvantages associated with autografts and synthetic scaffolds such as secondary surgery and uncertain quality and quantity of the graft material. However, a major drawback of allografts is its potential risk for disease transmission^4^. Therefore, the sterilisation process is the focus of allograft transplantation.

Allograft sterilisation aims to eradicate the survival of harmful organisms while maintaining important biological and structural properties of the grafts. Usually, tissue banks rely on aseptic procurement and processing that involves removal of debris and organic matter, soaking in various disinfectant solutions, and monitoring for microbes at various stages of processing. In addition, pretreatment including decellularisation to remove cellular antigens and reduce immunogenicity has the potential to eliminate the need for autologous or patient-matched grafts for direct clinical use^5^,^6^. However, terminal sterilisation is a critical step used to eliminate bacteria and viruses^5^,^7^. Currently, there is no general consensus on the method for terminal sterilisation of allografts. Some tissue banks have implemented secondary sterilisation methods such as low-to-moderate dose of gamma irradiation (GI).

Sterilisation of biological tissues can be problematic because these processes typically have an aggressive mechanism of action that can adversely affect the natural tissue properties. Alteration of the natural properties such as biomechanics, physical structure, and surface chemistry potentially mitigate the benefits of using naturally derived materials for tissue regeneration by modifying intrinsic factors that direct cell adhesion and tissue regeneration^8^,^9^,^10^,^11^.

In the current decade, GI is the most prevalent method of sterilisation used by tissue banks^12^. It is used as a gold standard to provide safety against disease transmission and is beneficial in eradicating human immunodeficiency virus and hepatitis C in bone allografts at a radiation level of 25–35 kGy^13^,^14^,^15^. However, the irradiation dose needed to achieve a sterility-assurance level of 10^−6^ is 89 kGy^13^. Sterilisation by GI occurs through the formation of free radicals through radiolysis of the water present in collagen, which can lead to post-implantation difficulties such as prefailure and/or secondary fractures of bone allografts^16^. Furthermore, higher doses of radiation can result in modifications of the microscopic and ultrastructural appearance of tendons and ligaments^17^. As such, it can result in a significant decrease in the mechanical strength of the graft, rendering it unsuitable for implantation^18^.

Some studies have reported that allografts become less efficacious following GI^19^,^20^,^21^. Several studies have also focused on the effect of GI on osteoinductive activities of allografts. Although GI has not been associated with chemical changes in soft tissue, it has been shown to produce structural and mechanical changes that may affect cellular interactions^22^,^23^. Bone allografts should be defatted to prevent cell death induced by peroxidised lipids. These peroxidised lipids are formed from lipids present in the medullary spaces and can release compounds toxic to osteoblast-like cells^24^. Various studies have assessed how sterilisation affects the biomechanical properties of tissue grafts. The effects of radiation on collagen fibres include direct scission of alpha polypeptide chains or crosslinking of polypeptide chains in the presence of free radicals^25^,^26^.

It is often necessary for the tissue to be stored for extended periods of time prior to the commencement of tests or applications^27^,^28^,^29^. Because maintenance of biomedical products in aqueous solution can cause significant product deterioration, preserved tissues are commonly dehydrated or frozen^30^. However, allografts are better preserved in solution before use, because organs of the human body have a high water content. Therefore, it is important to identify a method better than GI, which will not affect the biological and mechanical properties of the sterilised tissue and maintain the wet state of the allografts.

Polyvinylpyrrolidone-iodine (PVP-I) is an iodine-containing antiseptic compound used for a long time as a mucosal skin disinfectant^31^. It has useful properties for local anti-infective treatment, in particular, surface decontamination and has a broad microbicidal action spectrum including bacteria, fungi, protozoans, and viruses^32^. Furthermore, it can kill microbes in seconds with nearly no resistance and has minimal allergic and toxic potential^33^,^34^. Therefore, PVP-I is a promising candidate for many new applications such as wound healing, ocular treatment, inhalative treatment of the respiratory tract, intra-articular infections, and prophylaxis after joint surgery.

PVP-I solution enhances osteogenic differentiation in an osteoblast-like cell line, MC3T3-E1^35^. More recently, the biocompatibility and biological effects of PVP-I on cells from joint tissue were examined, and the results indicated the potential of PVP-I for osteochondral defect repair^36^. The main component of PVP-I is iodine and poly (N-vinyl-2-pyrrolidone) (PVP). PVP is a water soluble and low cytotoxic organic polymer, as well as a hydrophilic polymer excipient. As reported previously, osteogenic differentiation of BM-MSC was enhanced by PVP-I, but not by PVP, thus implying that the iodine molecule is the active ingredient^36^.

In our previous study, by *in vitro* and *in vivo* tests showed that demineralised bone matrix (DBM) sterilised by PVP-I could achieve enhanced osteogenesis compared with irradiated DBM^37^. Therefore, taking these findings forward, in this study, we aimed to determine whether PVP-I can be used for sterilisation, in particular, terminal sterilisation. Additionally, we assessed whether it can be used as a new wet-preservation method capable of maintaining the mechanical properties of tissue grafts while enhancing their osteogenesis as well as tried to identify its underlying mechanism of action.

## Results

### PVP-I has effective antimicrobial efficacy

**Figure 1:**
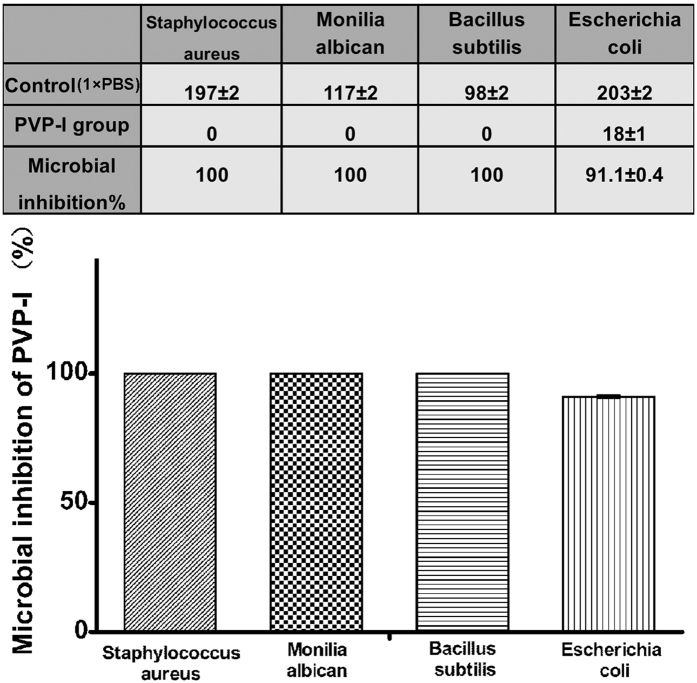
Efficacy of sterilisation by polyvinylpyrrolidone-iodine (PVP-I) on contaminated demineralised bone matrix (DBM) at 24 h. Numeration by colony counting shows 100% inhibition of selected gram-positive bacteria (*Staphylococcus aureus*), fungus (*Monilia albicans*), and bacterial endospores (*Bacillus subtilis*) and 91% inhibition of gram-negative bacteria (*Escherichia coli*).

In order to use PVP-I as a sterilising and preservation agent, we first tested its efficacy to inhibit microbial growth. Contamination was indicated by the growth of microbial colonies. The colony-counting method was used, and each result is presented as the average of 5 replicate assays. Numeration on model agar medium showed 100% inhibition of the growth of selected gram-positive bacteria (*Staphylococcus aureus*), fungus (*Monilia albicans*), and bacterial endospores (*Bacillus subtilis*) and 91% inhibition of the growth of gram-negative bacteria (*Escherichia coli*) (Fig. 1). PVP-I did not show 100% inactivation of gram-negative bacteria after 24 h of sterilisation of porous DBM contaminated in microbial/Dulbecco’s modified Eagle’s medium (DMEM) media. However, it showed excellent pathogen-inactivation ability and inactivated all the selected gram-positive bacteria, fungi, and bacterial endospores.

### PVP-I–preserved allografts enhance osteogenesis *in vivo*

**Figure 2:**
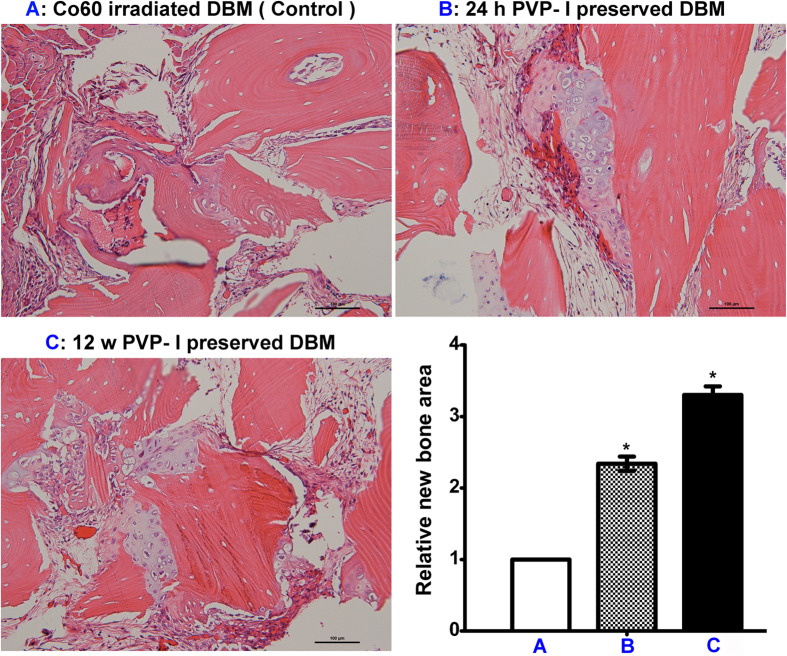
Haematoxylin-eosin (H&E) staining of histological sections of polyvinylpyrrolidone-iodine (PVP-I)–sterilised demineralised bone matrix (DBM) group and control group (Co_60_ sterilised). No difference is seen in the osteogenesis properties of PVP-I–preserved DBM at 24 h and 12 weeks (P = 0.618, P > 0.05). However, they both show better performance than Co_60_-irradiated DBM (P = 0.000, P < 0.05). The new-bone area in the PVP-I–sterilised DBM group is significantly larger than that in the Co_60_-irradiated DBM group. Further, PVP-I–preserved DBM induces better bone regeneration than the Co_60_-irradiated DBM. *P < 0.05. Scale bar = 100 μm.

We found in a preliminary study that implantation of PVP-I–preserved DBM induced better bone regeneration compared to Co_60_-irradiated ones in a mouse model (Fig. 2). Therefore, we aimed to analyse this finding in the current study. Compared with the Co_60_-irradiated DBM group, the new bone area was significantly larger in the PVP-I–sterilised DBM group (P = 0.000, P < 0.05). To test the effects of preservation time on osteogenesis, we extended the preservation time from 24 h to 12 weeks. There was no difference in the osteogenic properties of PVP-I–preserved DBM between these two time points (P = 0.618, P > 0.05), but both PVP-I–preserved DBM showed better results than Co_60_-DBM (P = 0.000, P < 0.05).

**Figure 3:**
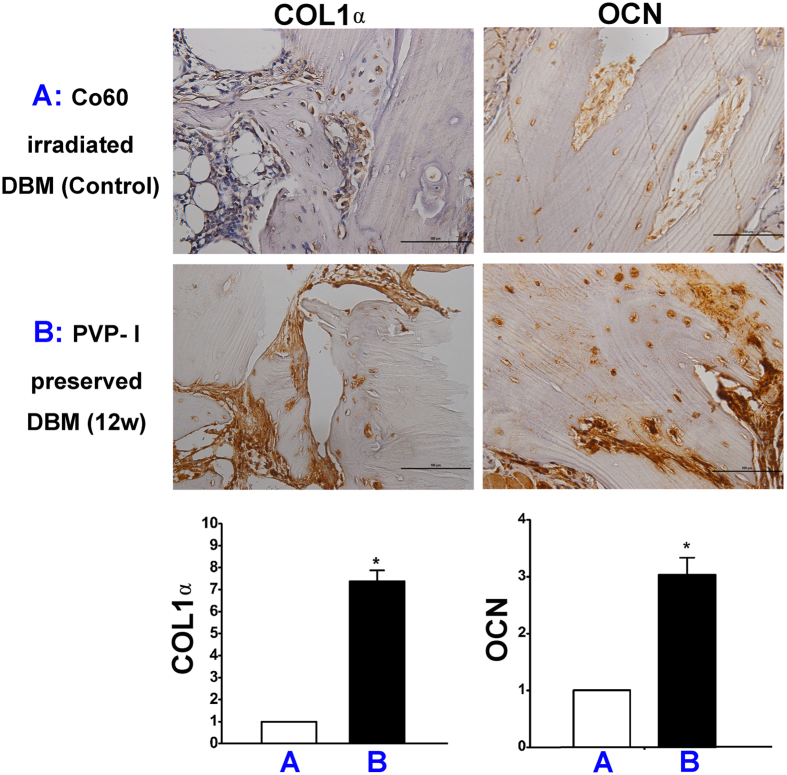
Immunohistochemical evaluation of Col-1α and OCN expression at the surgical sites. COL-1α and OCN expressions are significantly higher in the polyvinylpyrrolidone-iodine (PVP-I) group (preservation time of 12 weeks) than in the Co_60_-irradiated group (P = 0.000, P < 0.05). *P < 0.05. Scale bar = 100 μm.

The immunohistochemistry assay (preservation time of 12 weeks) suggested that COL-1α and OCN expression was significantly higher in the PVP-I group than in the Co_60_-irradiated group (P = 0.000, P < 0.05), which explains the better bone regeneration in the former group (Fig. 3). The images of preservation time 24 h were not shown because there was no difference between 24 h and 12 weeks.

### PVP-I–preserved allografts have better surface properties

**Figure 4:**
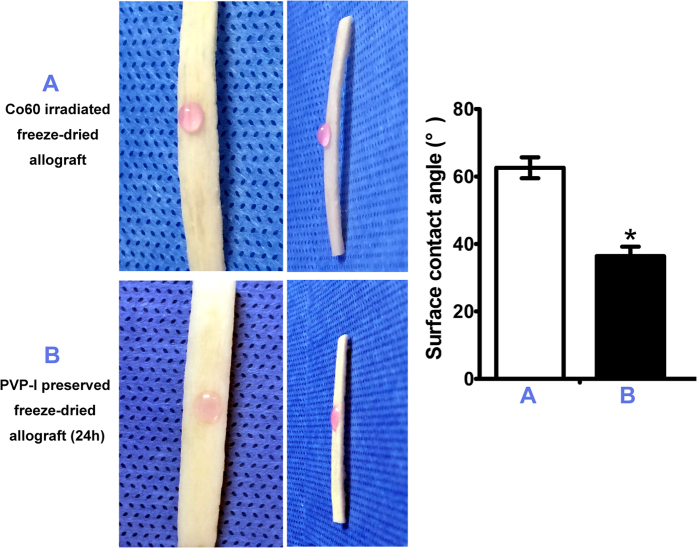
Surface contact angle detection of polyvinylpyrrolidone-iodine (PVP-I)–sterilised freeze-dried allografts and control group (Co_60_ sterilised). The two groups both show hydrophilic surfaces with contact angles less than 90°. However, the surface contact angle of PVP-I–sterilised freeze-dried allografts (preservation time of 24 h) is significantly lower than that of the Co_60_-irradiated group (P = 0.000, P < 0.05). *P < 0.05.

Wettability was determined by contact angle measurements. Hydration with α- minimum essential medium (α-MEM) demonstrated that both irradiated and PVP-I preserved samples (preservation time of 24 h) were hydrophilic as defined by a contact angle <90°. PVP-I preserved samples with contact angles averaging 36.4°were significantly more hydrophilic than irradiated ones that averaged 62.6° (P = 0.000, P < 0.05) (Fig. 4).

**Figure 5:**
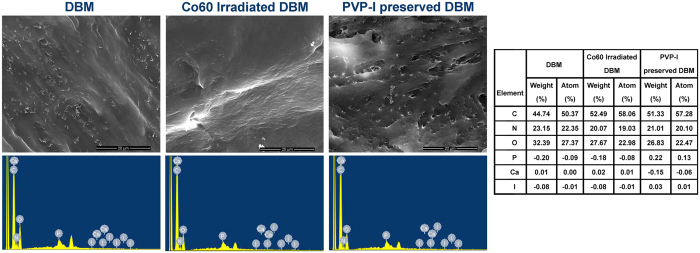
Surface morphology of DBM by scanning electron microscope (SEM) and surface elemental analysis by energy-dispersive X-ray spectroscopy (EDS). The surface morphology of polyvinylpyrrolidone-iodine (PVP-I)–sterilised demineralised bone matrix (DBM) (preservation time of 24 h) is clearly different from Co_60_ -irradiated one. The former one has a rougher surface with many open pores similar to the morphology before sterilization, while the latter exhibits a smooth and flattened surface. EDS measurements indicates that the three groups generally share a similar composition. However, trace amount of iodine can be detected on the surfaces of PVP-I–preserved DBM. Scale bar = 20 μm.

Scanning electron microscope (SEM) micrographs and energy-dispersive X-ray spectroscopy (EDS) results of the samples are shown in Fig. 5. The surface morphology of PVP-I preserved DBM (preservation time of 24 h) is clearly different from Co_60_ irradiated one. The former one has a rougher surface with many open pores, similar to the morphology before sterilization, while the latter exhibits a smooth and flattened surface. EDS measurements were performed to determine the elemental composition of the surface, with a particular focus on iodine content. The results indicated that the three groups generally share a similar composition. However, trace amount of iodine can be detected on the surfaces of PVP-I preserved DBM.

**Figure 6:**
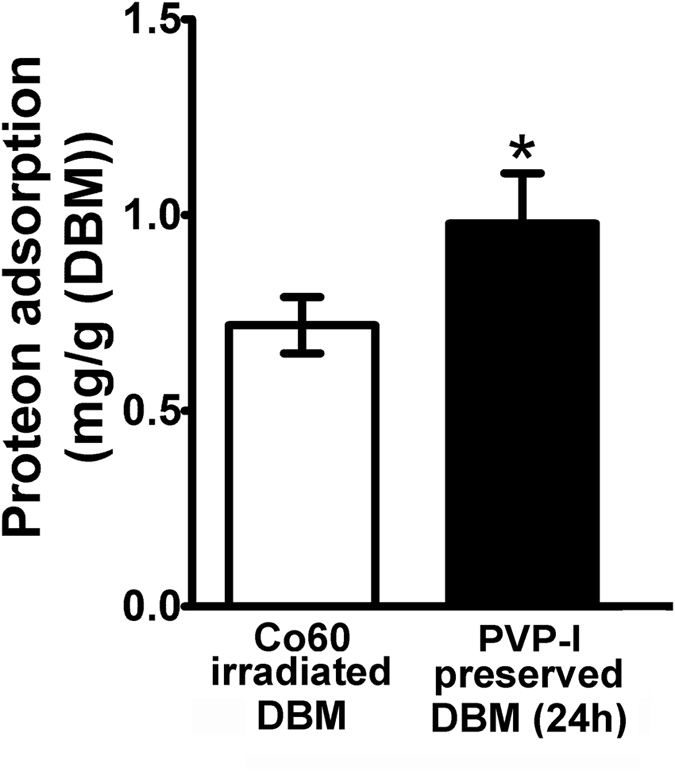
Protein adsorption ability test of polyvinylpyrrolidone-iodine (PVP-I)–sterilised freeze-dried allografts and control group (Co_60_ sterilised). Protein adsorption values are significantly higher in PVP-I group (preservation time of 24 h) than in the Co_60_-irradiated group (P = 0.0015, P < 0.05). *P < 0.05.

Protein adsorption behaviour onto the samples is plotted in Fig. 6. The results showed that PVP-I group (preservation time of 24 h) had a significant effect on protein adsorption. Its values were significantly higher than irradiation group (n = 6, P = 0.0015, P < 0.05).

### PVP-I–preserved allografts maintain better mechanical strength

**Figure 7:**
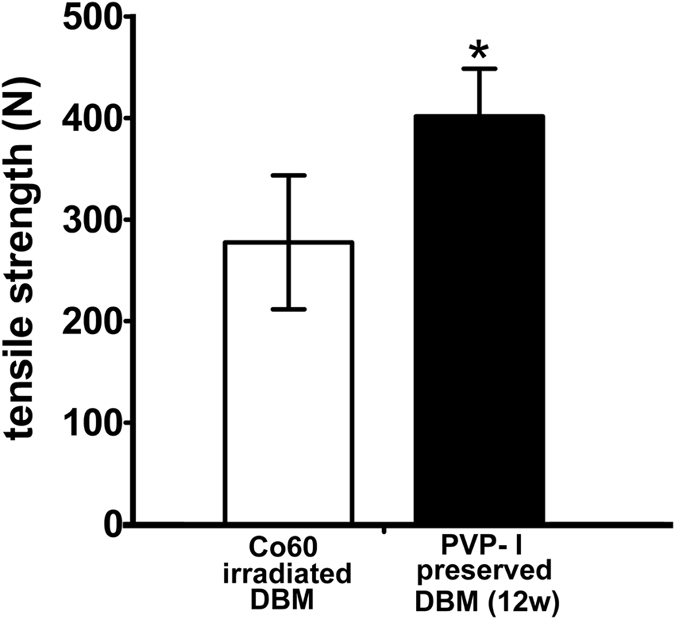
Biomechanical evaluation of tendons. The tensile strength of the tendon is 278.71 ± 66.01 N for the Co_60_-irradiated group (25 kGy) and 402.83 ± 46.90 N for the polyvinylpyrrolidone-iodine (PVP-I) group as compared to the Co_60_-irradiated group (P = 0.009, P < 0.05). PVP-I–preserved tendons show higher tensile strength than the Co_60_-irradiated tendons. *P < 0.05.

To clarify the effects of PVP-I preservation on the biomechanical properties of tendon, stress–strain measurements were conducted. The tensile strength of the tendons was 278.71 ± 66.01 N for the Co_60_-irradiated group (25 kGy) and 402.83 ± 46.90 N for the PVP-I group (preservation time of 12 weeks) (P = 0.009, P < 0.05). We found that PVP-I–preserved tendons showed higher tensile strength than the Co_60_-irradiated tendons (Fig. 7).

### PVP-I–preserved allografts contains residual iodine

**Figure 8:**
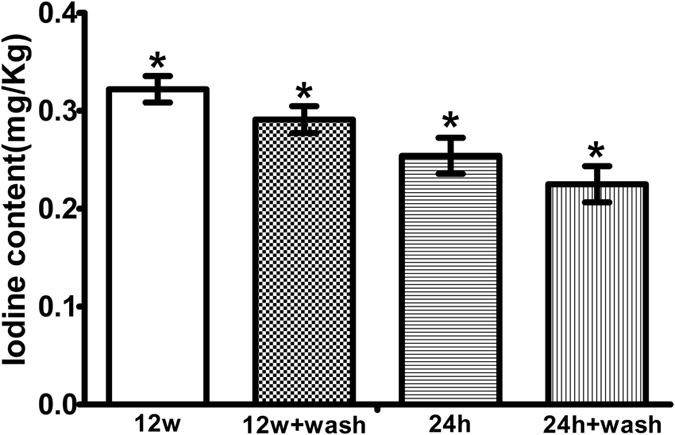
Iodine residue of polyvinylpyrrolidone-iodine (PVP-I)-preserved allografts. The iodine content is the highest in 12-week group and lowest in 24 h + wash group (12 weeks > 12 weeks + wash > 24 h > 24 h + wash). Significant differences are observed between any two of the four groups. (P = 0.002 for (12 weeks) and (12 weeks + wash), P = 0.003 for (24 h) and (24 h + wash), P = 0.000 for comparison between any two of the rest groups). *P < 0.05.

Residual iodine was detected in all groups in this study and its content was highest in the 12-week group and lowest in the 24 h + wash group (12 w > 12 w + wash > 24 h > 24 h + wash) (Fig. 8). Significant differences were noted between any two of the four groups (P = 0.002 for (12 w) and (12 w + wash), P = 0.003 for (24 h) and (24 h + wash), P = 0.000 for comparison between any two of the rest groups, P < 0.05).

### Appropriate concentration of PVP-I is cytocompatible and can stimulate osteogenic differentiation *in vitro*

**Figure 9:**
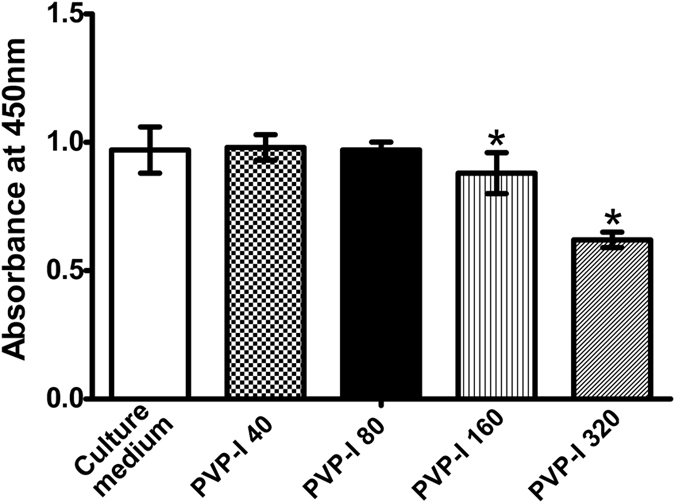
Cytotoxicity of polyvinylpyrrolidone-iodine (PVP-I) for MC3T3-E1 cells evaluated by CCK-8 assay. The CCK-8 assay shows cell proliferation when the concentration of PVP-I is >80 ng/mL (P = 0.043, P = 0.000, P < 0.05). The inhibitory effects are not evident when the concentration of PVP-I is ≤80 ng/mL (P = 0.787, P = 1.000, P > 0.05). Complete culture medium is used as the control. *P < 0.05.

Cell tests were used to verify the effects of PVP-I on pre-osteoblast cells, including their cytotoxicity and osteogenic properties. The Cell Counting Kit-8 (CCK-8) showed reduced proliferation of MC3T3-E1 cells when the concentration of PVP-I was >80 ng/mL (P = 0.043, P = 0.000, P < 0.05). This effect was eliminated and even slightly promoted when the concentration of PVP-I was ≤80 ng/mL (Fig. 9). However, this result was not significant (P = 0.787, P = 1.000, P > 0.05). Therefore, 80 ng/mL was chosen as the appropriate concentration for further differentiation tests.

**Figure 10:**
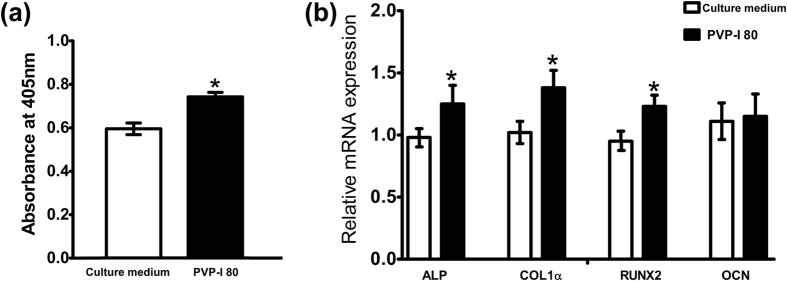
Detection of osteogenic markers. (**a**) Alkaline phosphatase (ALP) activities of MC3T3-E1 cells show a significant increase in the polyvinylpyrrolidone-iodine (PVP-I) group (P = 0.000, P < 0.05); (**b**) Expression of genes related to osteogenesis shows upregulated expression of osteogenic gene markers including *COL-1α, ALP*, and *RUNX2* in the PVP-I group (P = 0.044, 0.020, 0.016; P < 0.05). Complete culture medium is used as the control. *P < 0.05.

The alkaline phosphatase (ALP) concentration detected by the *p*-nitrophenyl phosphate (*p*NPP) method showed a significant increase in the PVP-I group compared with complete culture medium group (P = 0.000, P < 0.05) (Fig. 10a). Moreover, real-time quantitative polymerase chain reaction (RT-qPCR) showed upregulation of osteogenic gene markers including *COL-1α, ALP*, and *RUNX2* in the PVP-I group (P = 0.044, 0.020, and 0.016, respectively; P < 0.05) (Fig. 10b). However, the expression of *OCN* did not significantly increase on day 3 (P = 0.782, P > 0.05).

## Discussion

The basis of this study was the preliminary finding that *in vivo* bone formation of PVP-I–preserved allografts was better than Co_60_-irradiated allografts. Combined with the results reported previously, we hypothesised that PVP-I preservation may be a terminal method for sterilisation and preservation of allografts. This is the first study to report excellent performance of PVP-I–preserved allografts in tissue engineering and to explore its underlying mechanisms.

In order to prove the abovementioned hypothesis, we performed 4 steps. First, microbial inactivation tests were performed to determine the sterilisation efficacy of PVP-I. Second, an *in vivo* mice model was prepared to confirm bone formation in the PVP-I–preserved allografts. Third, we explored the underlying mechanism of action of PVP-I preservation. We thought the excellent osteogenic performance may come from different surface properties and residual iodine in PVP-I–preserved allografts. Therefore, surface morphology was observed. Contact angles and protein adsorption abilities were examined. And the content of the residual iodine was measured. Finally, we performed *in vitro* cell assays to detect the effects of PVP-I on cell proliferation and osteogenic differentiation.

PVP-I is widely used for lavation and irrigation during open joint surgery to prevent surgical site infection^38^,^39^,^40^. Its sterilisation efficacy is closely related to its concentration and can be increased with time^32^. Recently, Soyer J *et al*. have successfully used a 10% povidone-iodine solution to decontaminate inoculated bone grafts^41^. PVP-I used here is a commercially available product, which makes it easy for future clinic applications. To confirm the consistency between different manufacturers, we tested several different brands. The effective iodine concentration of all these products is 0.5% ± 0.05% (w/v). Its applications include to hand brushing before operation, skin disinfection before surgery and injection, mucous disinfection; and wound disinfection, etc. This is the only formula of PVP-I we used for sterilization and preservation DBM.

The inactivation of four kinds of microbes was investigated in this study. The pathogens tested included bacterial spores, fungi, and gram-negative and gram-positive bacteria with aerobic and anaerobic growth characteristics. The microorganisms were selected on the basis of clinical importance and occurrence as a contaminant in tissue products. We achieved complete inactivation of gram-positive bacterium, a spore, and a fungus as well as less-than-ideal, but acceptable, inhibition of gram-negative bacterium. These differences in inhibition performance may be a result of differences in cell membrane properties. We expected greater inactivation of gram-negative bacterium with a thorough rinse to remove the residual organic media in the porous DBM and a longer sterilisation time. However, as a terminal sterilisation method, the antibacterial ratio obtained was sufficient to achieve a sterility assurance level of 10^−6^. Therefore, PVP-I can be used as a terminal sterilisation method and produce safe allografts.

In the current study, PVP-I showed additional benefits besides excellent pathogen inactivation. We evaluated the mechanical properties of biological tissue, which is important for improving the standard of healthcare. PVP-I–treated and Co_60_-irradiated tendon allografts were prepared in similar size and shape, following which their mechanical properties were compared. Our results showed that the PVP-I–treated tendons were stronger. The difference in maximal load to failure was statistically significant and demonstrated that the PVP-I–treated tendon allografts were stronger than the Co_60_-irradiated allografts.

The biomechanical parameter of Co_60_-irradiated tendons showed a significant decline after irradiation, making it unsuitable for clinical application. This finding also validates the concern regarding the use of irradiated tendon allografts with decreased mechanical properties. There is a significant amount of literature documenting a dose-dependent, deleterious effect of conventional GI on the biomechanical properties of tissues^42^,^43^. Consequently, there is a general negative perception that high doses of GI adversely affect graft integrity and efficacy. Bone loses a significant amount of strength during compression, bending, and torsion when exposed to >30 kGy doses of conventional irradiation^42^,^43^,^44^. We used tendons for the test instead of bone grafts, because the strength of the former is more important in determining postoperative management. Our results proved that PVP-I is a better choice than GI for maintaining the mechanical properties of tendons. However, our study does not clarify whether the allograft ever attains the strength of non-treated or fresh allografts. Nevertheless, long-term studies are needed to identify the changes in the strength of PVP-I–preserved allografts and determine whether their strength is maintained after treatment with PVP-I or increases/decreases over time.

Then, we used *in vivo* mice model to detect the osteogenic properties of PVP-I. The nude mouse model was selected to prevent the immune system reaction of the animal, which will increase the likelihood that an observed response is not derived from the implants but immunogenic. Therefore, athymic animals are typically used by industry to assay osteoinduction ability of commercial human DBM products for this reason^45^. The only difference in the PVP-I treatment is the sterilization/preservation time. Currently, we tested two time points, 24 h and 12 weeks. We found that both PVP-I–preserved DBM induced better bone regeneration than Co_60_-irradiated DBM, which is consistent with the finding of Jiang YZ *et al*.^36^ who concluded that an appropriate concentration of PVP-I is good for bone regeneration. Our *in vivo* results also suggested that the activity of DBM is enhanced by PVP-I.

Quantitative comparison of new bone formation by assessment of haematoxylin-eosin (H&E)-stained images confirmed that there were no differences between the PVP-I–preserved allografts at 24 h and 12 weeks, indicating that PVP-I can be used to preserve allografts. Therefore, allografts preserved in PVP-I can be sterilised while achieving higher osteogenic bioactivity.

Immunohistochemical staining showed that expression of CoL-1α and OCN was upregulated in the PVP-I group compared with the Co_60_-irradiated group. This *in vivo* result is consistent with previously reported *in vitro* study results^36^, indicating the enhanced expression of the same osteogenic gene markers.

Different sterilization methods result in variations of the scaffold ultrastructure, mechanics, and chemistry that affected its performance *in vitro* and *in vivo*. Contact angle was tested by α-MEM to enhance the visibility. According to our SEM images, contact angle and protein adsorption ability results, the surface properties of PVP-I preserved allografts and Co_60_ irradiated ones were significantly different. The PVP-I method can preserve the original morphology of the allografts with hydrophilic surfaces. It showed a decrease in contact angle compared with irradiated ones. In contrast, irradiation made changes to the original surfaces by flattening them. The current results are consistent with previous report that radiation can result in modifications of the microscopic and ultrastructural appearance of allografts^17^. Hydrophilicity and wettability are closely related and an important parameter for adhesion of proteins and cells. Adsorption of protein to biomaterial surfaces may precede and facilitate adhesion of cells to implanted materials. The protein study results confirmed what was shown in SEM images that PVP-I preserved allografts had an increased surface area when compared with irradiated ones. So, we have good reasons to assume that there is a similarity to adhesive proteins like fibronectin, and thus imply the possible differences in adhesive protein adsorptions and the resultant initial cellular events. In all, the inferior performance of irradiated ones may partly come from the poor surface properties compared with PVP-I preserved ones.

Surface property is one of the most implant factors in scaffold bioactivities. Another is the incorporation of bioactive element. The iodine molecule is the active ingredient in PVP-I^36^. As indicated by EDS detection, there was a little iodine on the surfaces of PVP-I preserved allografts. Therefore, we further quantitatively measured the residual iodine concentration. It was suggested that a prolonged preservation with PVP-I can increase the residual iodine content and washing can decrease this content. However, the highest content in this study was still far below the safety concentration stipulated by the Chinese Standard GB 5461–2000, indicating that the iodine content of PVP-I–preserved allografts is safe for the human body.

The bioactive properties of DBM originate from bone morphogenetic protein (BMP). The effect of GI on the osteoinductivity of DBM is controversial, because gamma-irradiated DBM samples can also release BMP^46^. Therefore, BMP is not the main reason for enhanced osteogenesis of PVP-I compared to GI. In other words, the effect of iodine outweighs the effects of released BMP.

As expected, residual iodine was detected in PVP-I–preserved allografts. However, its concentration was very low, i.e., below micromolar level. When used as a sterilisation and preservation agent, a small amount of PVP-I remains in the grafts after thorough washing with saline. Moreover, the presence of blood and exudate can further dilute the concentrations of these antiseptics in an *in vivo* microenvironment. Thus, the real PVP-I concentration in the implanted grafts must be much lower than the concentration used. Care must be taken with the use of PVP-I, as it can have negative effects such as cytotoxicity. PVP-I has been used to disinfect zebrafish embryo^47^, but it is toxic to brain tissues^48^. Furthermore, exposure to PVP-I, the concentrations of which are even lower than those used clinically, is toxic to epithelial cells^49^.

A cytocompatibility assay was performed to determine the appropriate PVP-I concentration for preservation and sterilisation of the allografts. Given the dose-dependent effects of PVP-I on cellular behaviour, the balance of negative effects on the proliferation and positive effects on osteogenic differentiation need to be optimised. Within the limits of the current study, we found that PVP-I at a concentration of 80 ng/L did not inhibit the proliferation of pre-osteoblast cells. However, it inhibited cell proliferation at higher concentrations, and this finding is consistent with that of Yang ZJ *et al*.^36^ moreover, our working concentration (80 ng/L) for osteogenic detection was lower than that used in their study, indicating that PVP-I can enhance osteogenic differentiation at lower concentrations as well.

The ALP and type I collagen concentrations in cultured MC3T3 cells notably increased in the PVP-I group in a similar pattern; however, the concentrations of OCN did not increase. A previous study reported that expression of OCN is induced only after expression of ALP and type I collagen^50^.

Iodine is known to be an essential micronutrient that plays a crucial role in the healthy development of animals^51^. It is a bioactive element and can benefit osteogenesis by producing thyroid hormone. Therefore, the initiation of osteogenesis in the PVP-I–preserved grafts may be attributed to both systemic and local effects, which together result in the upregulation of RUNX2, OCN, ALP, and CoL-1α expression, presenting as enhanced osteogenesis.

It is important that any pathogen-inactivation method for allografts provide the greatest possible reduction in infectivity while preserving the biophysical integrity of the tissue. Aseptic processing in the tissue-bank industry is both expensive and prone to failure^52^, and gamma irradiation is associated with significant compromises in the biomechanical properties of tissue allografts as well as the generation of toxic lipid compounds^53^. In comparison, PVP-I is a relatively simple material that can be directly obtained from a drug store without prescription. Therefore, PVP-I may emerge as a more compatible option for increasing the bioactivity of human allograft tissue, which will increase the use of human allograft tissue in orthopaedic surgeries. Thus, the industry can benefit from new sterilisation technologies including preservation of the meniscus, tendon, and osteochondral allografts.

In our future studies, we plan to explore the detailed mechanism of this action of PVP-I and extend the preservation time. And we are planning to use *in vivo* bone defect model to confirm the osteogenesis of PVP-I treated DBM. The data obtained in the present study may serve as a baseline to illustrate the use of PVP-I as a method for tissue sterilisation and preservation.

In conclusion, allografts preserved in PVP-I solution present with improved mechanical property and osteogenesis. PVP-I does not do hamper the biological and mechanical properties of sterilised tissue and maintains the wet state of the allografts. The improved biological performance of PVP-I preserved allografts may be attributable to better surface properties and residual PVP-I. The current findings suggest that PVP-I immersion can be used as a simple and easy method for sterilisation and preservation of a variety of allografts.

## Methods

### Human materials

The demineralisation process of DBM was similar to that employed by Urist *et al*.^54^ with the addition of several wash steps to remove the residual chemicals. The particle size of used DBM varied from 125 to 850 microns, and the DBM underwent lyophilisation after processing to produce a dry final product. Co_60_-DBM was produced using 25 kGy irradiation. PVP-I–sterilised DBM was produced by immersing the DBM in PVP-I (0.5%, 40 mM).

### Antimicrobial effectiveness of PVP-I

According to the requirements of Chinese Standard GB15981–1995, 4 standard strains*—S. aureus* (ATCC 6538), *B. subtilis* (ATCC 9732), *E. coli* (ATCC 25922), and *C. albicans* (ATCC 10231)—were used to determine the effectiveness of PVP-I sterilisation. Two milliliters of each microbial solution at a concentration of 10^7^ CFU/mL was used to contaminate Co_60_-DBM (1 g) for 12 h at 4 °C. After a thorough rinse with sterilised saline, 2 mL of 0.5% PVP-I (ANNJET, Dezhou, China) was used to sterilise the contaminated samples at 4 °C for 24 h. The contamination controls were sterilised using phosphate-buffered saline (PBS) solution (1×) to test their initial contamination level. Thereafter, the samples were incubated with 10 mL DMEM (Gibco, Grand Island, USA) for 12 h at 37 °C under sterile conditions. At the end of the incubation period, the supernatant was collected and diluted 1,000 times for further use. Subsequently, 100 μL of diluted supernatant was mixed with 20 mL nutrient agar media at 45 °C and cooled to form solid ager on a plate (diameter, 60 mm). After culturing at 37 °C for 24 h, the colonies on the agar plate were counted by naked eye. Microbial inhibition was calculated using the formula below:

Microbial inhibition% = [(Colonies in control group − Colonies in PVP-I group)/Colonies in control group] × 100%.

### Measurement of residual iodine content

The PVP-I–preserved allograft samples were categorised into four test groups: allografts preserved for 24 h (24 h), allografts preserved for 24 h and thoroughly rinsed with sterilised de-ionised water (24 h + wash), allografts preserved for 12 weeks (12 w), and allografts preserved for 12 weeks and thoroughly rinsed with sterilised de-ionised water (12 w + wash). The iodine content was detected by inductively coupled plasma mass spectrometry (n = 9).

### Contact angle detection and protein adsorption ability test

The hydrophilic properties of PVP-I preserved freeze-dried allografts and Co_60_ irradiated ones were investigated. PVP-I preserved samples were thoroughly rinsed by 1 × PBS, and dried at 37°. The specimens (n = 9) were prepared by cutting them into strips. Surface contact angle measurements were done by dropping 10 μL α-MEM (HyClone, UT, USA) onto the top surface of each sample. A photograph was taken within 10 s. Each sample was repeated five times. Surface contact angles were measured by Image-Pro Plus 6.0, and an average value was obtained and analysed statistically.

The surface morphology was observed by a SEM (Qutan™ FEG 250, FEI, Oregon, USA). EDS was performed at the same time.

For protein adsorption, the procedure was performed as previously reported^55^. Briefly, samples were immersed in PBS for 2 h, and then immersed in 4.5 g/L bovine serum albumin (BSA, Sigma-Aldrich) solution at 37 °C for 24 h. Then rinsed with fresh PBS, and immersed in a solution of 1% sodium dodecylsulfate (SDS) in PBS and sonicated for 20 min to detach the BSA adsorbed on the samples. A protein analysis kit (micro BCA protein assay kit, Fisher Scientific, Pittsburgh, PA) was used to determine the BSA concentration in the SDS solution. The absorbance at 562 nm was measured via a microplate reader (Multiskan™ FC Microplate Photometer, Thermo Fisher Scientific, MA, USA). Standard curves were prepared using the BSA standard.

### Cell culture and seeding

The MC3T3-E1 osteoblast-like cells (Chinese Science Academy, Shanghai, China) used in our experiments underwent 6–11 passages. Cells were seeded on the samples at densities of 2 × 10^4^/mL in α-MEM (HyClone, UT, USA) containing 10% fetal bovine serum (FBS; HyClone) and 1% penicillin/streptomycin (Gibco, Grand Island, USA). Subsequently, the cells were cultured in an incubator at 37 °C at a humidity of 95% with 5% CO_2_. Totally, 100 μL/well was used for 96-well culture plates, and 2 mL/well was used for the 12-well culture plates.

### Cell viability assay

Commercial PVP-I solution was diluted by complete culture medium. The effective iodine content from PVP-I was set at 320 ng/mL, 160 ng/mL, 80 ng/mL, and 40 ng/mL. Complete culture medium was used as the control. Cell viabilities were measured after culturing the cells for 48 h at 37 °C in 96-well plates using a CCK-8 (Dojindo, Japan). After 2 h, the absorbance was recorded in each well using an automatic enzyme-linked immunosorbent assay reader (Multiskan™ FC Microplate Photometer, Thermo Fisher Scientific, MA, USA) at 450 nm.

### Measurement of ALP activity

MC3T3-E1 cells were incubated in a 96-well culture plate for 24 h at 37 °C. The effective iodine content from PVP-I was 80 ng/mL. Complete culture medium was used as the control. ALP activity was measured after 7 days of culture using the pNPP method (Meilun, Dalian, China) with the (Multiskan™ FC Microplate Photometer, Thermo Fisher Scientific, MA, USA). The absorbance was determined at 405 nm, and measurements were performed in triplicate and repeated at least three times. The result of ALP was normalized against total protein content measured by Bicinchoninic acid (BCA) method.

### RT-qPCR

MC3T3-E1 cells were incubated in a 12-well culture plate for 72 h at 37 °C. The effective iodine content from PVP-I was 80 ng/mL, and the complete culture medium was used as the control. mRNA expression of *CoL-1α, ALP, RUNX2*, and *OCN* were measured. Briefly, total cellular RNA was obtained using TRIzol reagent (Invitrogen, CA, USA) following the manufacturer’s protocol. After dissolution in DEPC-treated water (Ambicon, Inc., TX, USA), the concentration and purity of the RNA samples were determined using a NanoVue Plus (GE Healthcare, NY, USA) at 230, 260, and 280 nm. Complementary DNA (cDNA) was generated from the extracted total RNA by using a PrimeScript RT Master Mix kit (Takara Bio Co., Ltd, Tokyo, Japan). The primers used were designed by Shanghai Sangon Biotech Co., Ltd., (Shanghai, China) (Table 1), and glyceraldehyde-3-phosphate dehydrogenase (GAPDH) was used as an endogenous control for calculating differences in RNA levels by the 2^−ΔΔCT^ method. Real-time quantification of cDNA targets was performed using a RT-PCR System (7500-Fast; Applied Biosystems, CA, USA) with an UltraSYBR Mixture (Low ROX) (CWBiotech Co., Ltd., beijing, China). The reaction mix (50 μL) contained 50ng total cDNA, 0.2 μM of each primer, and appropriate amounts of enzymes and fluorescent dyes, as recommended by the supplier. The two-stage PCR program was performed as follows: 10 min at 95 °C for DNA polymerase activation, 40 cycles of 15 s at 95 °C, and a 1 min extension step at 60 °C. The PCR reaction was followed by melting-curve analysis to verify the specificity and identity of the RT-PCR products; this analysis can distinguish specific PCR products from nonspecific PCR products resulting from primer-dimer formation. The resulting data were analysed using the software provided by the manufacturer.

### Surgical procedures

For the surgical studies, PVP-I DBM was immersed in PVP-I for 24 h or 12 weeks at 4 °C. Co_60_-irradiated DBM sterilised with 25 kGy Co_60_ was used as the control. The osteoinductivity of PVP-I DBM and Co_60_-irradiated DBM was analysed using an *in vivo* ectopic pouch model of anesthetised BALB/c nude mice (1.5 month, male, 21 g ± 4 g, n = 8) (Animal Center of First Affiliated Hospital of CPLA General hospital, China). Samples (0.1 g each) were implanted per animal, and the animals were anesthetised via celiac injections. After thoroughly rinsing with sterilized saline, the samples were implanted into the gastrocnemius muscle of the hind limb using nude mice. Bilateral incisions in the hind limbs were made (longitudinal and 2 mm in length). The left side was used as the control site (implant of Co_60_ DBM), and the right site was used as the experimental site (implant of PVP-I DBM). Muscle pouches were closed by a single suture, which also served to secure the implant and locate the implantation site at the time of sacrifice. Intramuscular injection of antibiotics was administered post-surgery until wound healing. Wound clips were used to close the skin incisions, which were removed 7–10 days later. Animals were euthanized 28 days later by excessive inhalation of carbon dioxide.

### Histological and immunohistochemical analyses

The implanted DBM with its surrounding tissue was taken. The obtained samples were prepared for histological and immunohistochemical analyses.

For histological analysis, H&E staining was used to detect the host-implant reactions and new-bone formation. The samples were fixed in 4% paraformaldehyde for 24 h, decalcified in 10% EDTA for 2–4 weeks, embedded in paraffin, and cut using an RM2145 microtome (Leica, Solms, Germany) to yield 5-μm sections. The sections were then stained with H&E and evaluated under a light microscope (OLYMPUS BX50, OLYMPUS, Tokyo, Japan).

Immunohistochemical analysis of ALP and osteopontin (bone-specific protein) was performed by incubating the samples with rabbit anti-mouse COL-1α antibody and rabbit anti-mouse OCN antibody (Abcam, Cambridge, England) overnight at 4 °C. The samples were then incubated with a secondary antibody (SA1052; Boster, Beijing, China). The images were visualised using the same optical microscope.

Images were captured by Nikon DS-US camera, and analyzed by Nis-Elements. Briefly, four randomly-chosen images for each sample were analyzed with eight samples per condition, yield 32 images each group.

### Biomechanical testing

Uniaxial tensile tests were performed using an all-purpose mechanical testing machine (MTS793; 0.05 mm/s, n = 5). The tested tendon was 170 mm in length and 3–5 mm in diameter.

### Ethics statement

The DBM and tendon used for this study was provided by the Tissue Bank of First Affiliated Hospital of China People’s Liberation Army (CPLA) General Hospital (http://www.304guke.com/zzk/zzkjj/88.html), which has a formal consent process. The donors provided documented consent for donating the tissue samples after their death. The authors of this study had no access to the information that would identify donor patients associated with the bone tissue samples.

The animal research was approved by the Animal Care and Use Committee (IACUC) of Institute of Orthopedics, First Affiliated Hospital of CPLA General hospital. The entirety of this specific study, including all of the intervention procedures and the euthanasia, was approved by the IACUC. All procedures were carried out in accordance with the approved guidelines. Informed consent was obtained from all subjects for human tendon samples.

### Statistical analysis

For quantitative tests, data are expressed as the mean ± standard deviation and were analysed using SPSS version 22 (SPSS, Chicago, IL). Tukey’s test was used for post-hoc multiple comparisons. A value of P < 0.05 was considered statistically significant.

## Additional Information

**How to cite this article**: Zhao, Y. *et al*. Use of polyvinylpyrrolidone-iodine solution for sterilisation and preservation improves mechanical properties and osteogenesis of allografts. *Sci. Rep.* **6**, 38669; doi: 10.1038/srep38669 (2016).

**Publisher's note:** Springer Nature remains neutral with regard to jurisdictional claims in published maps and institutional affiliations.

## Figures and Tables

**Table 1:** Real-time reverse transcription polymerase chain reaction (RT-PCR) Primers Used in This Study.

Target Gene	Forward primer (5′-3′)	Reverse primer (5′-3′)
RUNX2	5′-CCTCTGACTTCTGCCTCTGG-3′	5′-ATGAAATGCTTGGGAACTGC-3′
OCN	5′-GCCCTTAGCCTTCCATAGTG-3′	5′-CGGGATACACACACCCTCTT-3′
COL1 α	5′-TGACTGGAAGAGCGGAGAGT-3′	5′-GACGGCTGAGTAGGGAACAC-3′
ALP	5′-AAGCTGGGAAGAACACTCCA-3′	5′-CAAACAGGAGAGCCACTTCA-3′
GAPDH	5′-AGCAGTCCCGTACACTGGCAAAC-3′	5′-TCTGTGGTGATGTAAATGTCCTCT-3′
